# Unraveling the socio-cognitive consequences of KCC2 disruption in zebrafish: implications for neurodevelopmental disorders and therapeutic interventions

**DOI:** 10.3389/fnmol.2024.1483238

**Published:** 2024-10-14

**Authors:** Mohammad Naderi, Thi My Nhi Nguyen, Christopher Pompili, Raymond W. M. Kwong

**Affiliations:** Department of Biology, York University, Toronto, ON, Canada

**Keywords:** K^+^/Cl^–^ cotransporter 2, zebrafish (*Danio rerio*), memory, oxytocin, excitation inhibition balance

## Abstract

During postnatal brain development, maintaining a delicate balance between excitation and inhibition (E/I) is essential for the precise formation of neuronal circuits. The K+/cl− cotransporter 2 (KCC2) is instrumental in this process, and its dysregulation is implicated in various neurological disorders. This study utilized zebrafish (*Danio rerio*) to investigate the socio-cognitive consequences of KCC2 disruption. Through CRISPR-Cas9 technology, biallelic *kcc2a* knockout zebrafish larvae were generated, revealing behavioral abnormalities, including impaired social interactions and memory deficits. Molecular analyses unveiled alterations in key genes associated with the GABAergic and glutamatergic systems, potentially contributing to E/I imbalance. Additionally, KCC2 disruption influenced the expression of oxytocin and BDNF, crucial regulators of social behaviors, synaptic plasticity, and memory formation. The study also explored the therapeutic potential of KCC2 modulation using pharmaceuticals, showing the rescuing effects of CLP-290 and LIT-001 on social abnormalities. However, the selective impact of LIT-001 on social behaviors, not memory, highlights the complexity of neurobehavioral modulation. In summary, this study sheds light on the pivotal role of KCC2 in shaping socio-cognitive functions and suggests potential therapeutic avenues for KCC2-related neurological disorders.

## 1 Introduction

A delicate equilibrium between excitation and inhibition (E/I) of neurons and neuronal networks is crucial for the normal functioning of the central nervous system (CNS), ensuring precise neural communication and information processing (Buzsáki et al., 2007; Sohal and Rubenstein, 2019). The K^+^/Cl^−^ cotransporter 2 (KCC2/SLC12A5) is an evolutionarily conserved neuron-specific cation-chloride cotransporter that plays a vital role in establishing and maintaining this balance. KCC2 maintains neuronal chloride homeostasis and determines the hyperpolarizing activity of *γ*-aminobutyric acid A (GABA_A_) receptors. GABA exhibits an excitatory role during early brain development, transitioning to an inhibitory neurotransmitter in the mature brain. This dynamic nature of GABAergic transmission arises from alterations in the chloride (Cl^−^) gradient (Kaila et al., 2014). During the initial stages of brain development, the Na^+^–K^+^– Cl^−^ cotransporter 1 (NKCC1) predominates, facilitating the intracellular accumulation of chloride ions. Subsequently, the activation of GABA_A_Rs triggers the efflux of Cl^−^ ions, thereby inducing membrane depolarization. As the nervous system matures, up-regulation of the neuronal chloride extruder KCC2 and down-regulation of NKCC1 cause a progressive reduction of intracellular Cl^−^ concentration in neurons, resulting in a hyperpolarizing shift of Cl^−^ reversal potential and an excitation-to-inhibition switch of GABAergic action (Ben-Ari et al., 2007). Besides the chloride extrusion function, KCC2 promotes functional maintenance and plasticity of glutamatergic synapses (Li et al., 2007; Gauvain et al., 2011; Chevy et al., 2015). In line with its pivotal role in regulating inhibitory and excitatory neurotransmission, alterations in KCC2 expression and function have emerged as a common mechanism underlying a range of human brain disorders, including epilepsy (Puskarjov et al., 2014), schizophrenia (Hyde et al., 2011), Rett syndrome (Banerjee et al., 2016; Hinz et al., 2019), and autism spectrum disorders (ASD) (Merner et al., 2015). Likewise, studies on rodent models have revealed that alterations in KCC2 function are associated with Downs syndrome, fragile X syndrome (Tyzio et al., 2014), Rett syndromes (El-Khoury et al., 2014), and ASD (Anacker et al., 2019). The critical role of KCC2 in early brain development underscores its potential as a target for innovative therapeutic strategies. Consequently, there has been a surge of research on therapeutic strategies to safely upregulate KCC2 expression to promote neural inhibition (Duy et al., 2020; Tang, 2020).

KCC2 exists in two isoforms, KCC2a and KCC2b, differing by their N-terminal sequences but possessing comparable ion transport activity. The mRNA levels of the two isoforms are similar during the neonatal period. While KCC2b expression increases steeply across postnatal development, the overall expression of KCC2a remains relatively constant and then decreases to contribute only a part of the total KCC2 in the mature brain. This points to overlapping roles of KCC2a and KCC2b in neonatal neurons but presumably different roles in mature neurons (Uvarov et al., 2007; Markkanen et al., 2014). There is a growing body of evidence pointing to the multifaceted impact of KCC2 in the brain and its significance in shaping complex behaviors and social interactions. Rodent studies have shown that partial reduction and/or conditional deletion of KCC2 (both isoforms) led to spatial and nonspatial memory impairments, intracellular chloride accumulation, increased anxiety-like behaviors, seizure susceptibility, and hyposensitivity to thermal and mechanical stimuli but normal locomotor activity and motor coordination (Delpire and Mount, 2002; Tornberg et al., 2005; Kreis et al., 2023). KCC2b heterozygous knockout mice also exhibit altered social dominance behaviors and increased amplitude of spontaneous postsynaptic currents in the medial prefrontal cortex (PFC) (Anacker et al., 2019). While these studies have provided valuable insights into the functional roles of KCC2 and its impact on neural development and behavior, there is still a need for further investigation into the specific contributions of KCC2a and KCC2b isoforms in sociocognitive functions. Particularly, the precise role of KCC2a in regulating neurobehavioral functions remains elusive and warrants additional research.

The zebrafish (*Danio rerio*) has emerged as a promising model for unraveling the complexities of brain disorders, including attention-deficit/hyperactivity disorder (ADHD), schizophrenia, and particularly ASD. Its genetic manipulability and inherent neurodevelopmental traits, which resemble those found in humans, offers a refreshing departure from the conventional reliance on rodent models (Norton, 2013; De Abreu et al., 2020). By leveraging these traits, zebrafish provide a valuable tool for studying the complexities and dynamics of human brain disorders. In recent years, the combined utilization of zebrafish, along with state-of-the-art genomic editing technologies like the CRISPR/Cas9 (Clustered regularly interspaced short palindromic repeats (CRISPR)/CRISPR-associated protein 9) system, has emerged as a highly promising and efficient approach to accelerate the development of disease-relevant models, validate novel drug targets, and explore potential therapeutic interventions with significant clinical implications (Cornet et al., 2018). The KCC2-mediated GABA switch in zebrafish retinal neurons occurs at around 2.5 days post-fertilization (dpf) (Zhang et al., 2010; Zhang et al., 2013). Moreover, dysregulation of KCC2 expression impairs neural development and locomotor behaviors in zebrafish embryos (Reynolds et al., 2008), revealing a structural role of KCC2 in brain development. In spite of this, the potential long-term pathophysiological effects of embryonic KCC2 dysregulation on social behaviors and memory in zebrafish have yet to be studied. Thus, this study aimed to characterize the behavioral phenotype of CRISPR-Cas9/pharmacological induced-KCC2-deficient zebrafish as well as to determine the therapeutic potential of KCC2 modulation in biallelic F0 *kcc2a* knockout (KO) animals (hereafter referred to as crispants).

## 2 Materials and methods

Adult zebrafish (Tübingen Longfin strain) were reared at the York University aquatic facility in a recirculating system (Aquaneering, USA) under a 14:10 h light/dark cycle. Temperature was maintained at 28 ± 1°C, conductivity between 650 and 750 μS, pH at 7.4, and hardness (as CaCO3) at 150 mg/L. The zebrafish were fed twice daily with a combination of a commercial zebrafish diet (Zeigler, USA) and nutritious brine shrimp (*Artemia salina*). For breeding, females and males were paired in a 2:1 ratio in breeding tanks overnight. The following morning, eggs were collected and transferred to 90 mm Petri dishes.

### 2.1 Generation of KCC2a crispants

In this study, F0 knockout larvae for a single gene were generated using a protocol based on Kroll et al. (2021). The method involved the use of synthetic gRNAs, targeting three loci per gene, and adjusting the concentrations of total gRNA and Cas9 for injections. Specific crRNAs (Alt-R™ CRISPR-Cas9 crRNA, 1 μL) were selected from the Integrated DNA Technologies (IDT) database based on predicted efficiency, and then annealed with tracrRNA (Alt-R CRISPR-Cas9 tracrRNA, 1 μL) at 95°°C for 5 min to form the gRNA. In this study, we designed 3 distinct crRNAs targeting either exon 2, 3, or 4 on *slc12a5a* (*kcc2a*). The crRNA selection prioritized distinct exons, and the ranking followed the best predicted crRNA from the IDT database. The gRNA/Cas9 ribonucleoprotein (RNPs) complex was then prepared by incubating Cas9 protein (Alt-R S.p. Cas9 Nuclease V3, 57 mM, IDT) with equal volumes of gRNA at 37°°C for 5 min. The three RNP solutions were pooled in equal amounts before injections, and approximately 1 nL (~ 28.5 fmol of RNP per gene) of the pool was injected into the yolk at the single-cell stage using a Pneumatic PicoPump (SYS-PV830; World Precision Instruments, USA). To account for possible influence from the microinjection procedure, pooled RNP complexes were prepared from three scrambled crRNAs (IDT: Alt-R CRISPR-Cas9 Negative Control crRNA #1, 1072544; #2, 1072545; #3, 1072546) and were injected as described above. These embryos were used as controls for comparison with the KO mutants. This method provided a highly efficient approach for rapidly screening the functional involvement of *kcc2a* in zebrafish behavior and other complex phenotypes in the F0 generation. The details of the selected crRNAs (sgRNAs) and targeted loci can be found in Supplementary Table S1. Injected embryos were collected at 1 dpf and genomic DNA was extracted by incubating each embryo with 50 μL of 50 mM NaOH at 95°C for 10 min. After cooling to 4°C, 5 μL of 1 M Tris–HCl (pH 8) was added for neutralization. The PCR reaction mixture contained 10 μL of 5X buffer, 1 μL of 10 mM dNTP, 1 μL of 10 μM forward and reverse primer each, 0.25 μL of Tag Polymerase, and 36.5 μL of H_2_O. The PCR program was: 95°C for 3 min; then 40 cycles of: 95°C for 30 s, 60°C for 30 s, 72°C for 30 s; then 72°C for 5 min; 4°C hold. After PCR, the PCR products were run on a 3% (w/v) agarose gel to confirm the correct amplification of the target region. The PCR amplicons were purified using a DNA purification kit (Qiagen, Santa Clarita, CA) as per instructions. The clean PCR products were sent for Sanger sequencing (The Centre for Applied Genomics, the Hospital for Sick Children, Toronto). The sequence chromatograms and results were analyzed using SnapGene software (Supplementary Figure S1). The primer sets used for genotyping are listed in Supplementary Table S2.

### 2.2 Chemical exposure

A multifaceted validation strategy was undertaken to substantiate the efficacy of KCC2 gene deletion/disruption and comprehensively assess resulting phenotypic alterations. To this end, a group of wild-type zebrafish embryos was subjected to exposure to a KCC2 inhibitor VU0240551 (Cayman Chemical, USA). This pharmacological exposure sought to elicit effects similar to those expected in KCC2-mutant animals, thereby corroborating the functional impact of KCC2 disruption. In a complementary approach, another group featured wild-type zebrafish embryos was exposed to valproic acid (VPA, Sigma–Aldrich, USA), an anti-epileptic drug that is commonly used to induce ASD-like traits in various animal models, including zebrafish (Mabunga et al., 2015). This group served as a critical reference point, facilitating a comprehensive evaluation of whether *kcc2a* gene deletion effects were akin to those observed in established models of ASD. The stock solutions of VU0240551 and VPA were prepared by dissolving them in dimethyl sulfoxide (DMSO; Sigma-Aldrich, USA) and stored at −20°C until use. The working solutions of these chemicals were subsequently prepared by diluting the stock solution in embryo water to achieve the desired concentration. At 8 h post-fertilization (hpf), embryos were randomly distributed in wells (30 embryos per well) of a 6-well plate containing either VPA (10 μM), VU0240551 (500 nM), or DMSO (from 8 hpf to 5dpf). Exposure concentrations were chosen based on a preliminary range-finding test, with VPA ranging from 1 μM to 50 μM and VU0240551 ranging from 250 nM to 5 μM. The EC50 values were determined via behavioral and morphological assessments at 6 dpf, ensuring low mortality and minimal morphological abnormalities (data not shown).

In another scenario and in order to assess KCC2 as a potential therapeutic target in neurodevelopmental disorders, *kcc2a* crispants were treated with either CLP-290 or LIT-001 through bath immersion. CLP-290 is a novel KCC2-selective activator which effectively restores KCC2 expression in various pathological conditions (Gagnon et al., 2013; Lam et al., 2023). LIT-001 is a novel characterized nonpeptide oxytocin receptor (OXTR) agonist that improves social interaction in neurodevelopmental models of ASD and schizophrenia (Frantz et al., 2018; Piotrowska et al., 2024). Oxytocin (OXT) directly modulates KCC2 expression/stabilization at the plasma membrane during early windows of development (Leonzino et al., 2016). Therefore, by utilizing LIT-001, this study aimed to capitalize on oxytocin’s direct modulation of KCC2, exploring its therapeutic potential for neurobehavioral abnormalities. To this end, zebrafish larvae were subjected to either synthetic CLP-290 (25 μM) or LIT-001 (5 μM), in beakers containing 25 mL of each solution for 48 h (1 h/2 times per day). The control group was treated with 0.01% DMSO. Subsequently, larval zebrafish were washed 3 times with system water for 15 min before engaging in behavioral paradigms. CLP-290 and LIT-001 concentrations and exposure durations were selected based on prior research demonstrating a positive impact of two-day exposure to KCC2-enhancing drugs on ASD-like behaviors in zebrafish (Rahmati-Holasoo et al., 2023). The chosen concentrations were determined to prevent abnormal locomotor patterns and erratic swimming behavior.

### 2.3 Behavioral paradigms

#### 2.3.1 Social behaviors

Social deficits are core symptoms of ASD. Shoaling and social preference tests are two robust measures of social behaviors in zebrafish. Shoaling test was carried out at 21 dpf in a white custom made square shaped arena (15 cm × 15 cm) for 8 min (8 larvae per arena, 3 replicates per group, *n* = 4–7). The shoaling parameters included time spent in proximity (within 0.5 cm of another subject) and the inter-individual distance among a group of zebrafish. Social preference test in 21 dpf larvae was performed in custom-built behavioral setups as described elsewhere (Naderi et al., 2022). The arena consisted of three parts: a center area, an empty side, and a conspecific side (16 cm L × 6 cm W × 6 cm H). Adjacent to the conspecific side was a chamber (4 cm L × 6 cm W) with 5 stimulus zebrafish larvae matched in size to the focal fish. Zebrafish were allowed to explore the arena for 10 min. Social preference was measured as the percentage of total time spent in the conspecific zone (n = 29–30).

#### 2.3.2 Object recognition memory

Object-recognition memory in zebrafish larvae (21 dpf) was assessed based on their tendency to explore novel objects as described previously (Naderi et al., 2022). Zebrafish larvae were first habituated to an empty apparatus (square-shaped maze: 9 cm L × 9 cm W × 6 cm H) for 5 min twice daily over 5 consecutive days, spanning from 16 to 20 dpf. At 21 dpf, the subjects explored two identical objects for 8 min (two red round-shaped LEGO®). After a 1.5-h interval, one of the objects was replaced with a new item (a green round-shaped LEGO®), and the fish was allowed to explore the objects for another 8 min. The exploration ratio, calculated as time spent with the novel object divided by the total exploration time, determined the preference (*n* = 25–28). A score greater than 0.5 indicated a preference for the novel object.

#### 2.3.3 Whole-brain mRNA expression analysis by digital droplet PCR

Total RNA was extracted from whole-brains of zebrafish larvae (*n* = 5–7, with each replicate comprising a pool of 5 brains) using the RNeasy Mini Kit (Qiagen, Canada), followed by assessing RNA quality using a plate spectrophotometer (Take3, Biotek Synergy LX, USA). Subsequently, cDNA was synthesized from 1 μg of total RNA using the iScript cDNA synthesis kit (Bio-Rad, USA). Droplet digital PCR (ddPCR) was performed using EvaGreenTM supermix (Bio-Rad, USA) on the QX200 Droplet Digital PCR system (Bio-Rad, USA) to determine the absolute mRNA expression levels of target genes. After amplification, droplets were analyzed using the QX200 droplet reader and QuantaSoftTM software (Bio-Rad, USA). The mRNA expression of each target gene was normalized to the transcript levels of ribosomal protein L13a (*rpl13a*) and ribosomal protein S18 (*rps18*). Specific primers used in this study are listed in Table S3.

#### 2.3.4 Enzyme-linked immunosorbent assay

Zebrafish isotocin (IT) exhibits a significant resemblance to mammalian oxytocin. Consequently, IT levels in the brains of zebrafish larvae were quantified using the DetectX® Oxytocin Enzyme Immunoassay Kit (K048-H1, Assay Designs, Ann Arbor, USA), following the manufacturer’s guidelines. Each assay utilized a pooled sample of 6–8 brains (*n* = 4–6) that had been pre-treated with a protease inhibitor (Thermo Scientific, USA).

#### 2.3.5 Western blotting

Total proteins were extracted from a pool of 10 larval zebrafish brains in RIPA buffer (Thermo Fisher Scientific, USA). After 30 min of incubation at 4°C, homogenization was performed using TissueLyser II (Qiagen, USA) and cell debris was removed by centrifugation at 14,000 g for 20 min. The supernatant was collected and protein concentrations were quantified using a BCA protein assay kit (Thermo Scientific, USA). Protein samples (30 μg) were then heated at 95°C for 10 min and separated on a 12% polyacrylamide gel through SDS-PAGE. The proteins were then transferred to a polyvinylidene difluoride (PVDF) membrane using a BioRad Trans Blot Turbo (Bio-Rad, USA). After transfer, the membranes were stained with 0.1% Ponceau S (dissolved in 5% acetic acid) and visualized on an iBright Imaging System (Invitrogen, USA) for subsequent normalization to total protein. Following this, the membrane was blocked with 5% non-fat milk in TBST (10 mM Tris pH 8.0, 150 mM NaCl, 0.5% Tween 20) for 2 h at room temperature followed by overnight incubation with anti-BDNF antibody (1,400, Invitrogen, USA) at 4°C. After washing with TBST for 20 min, the membranes were further incubated with a goat anti-rabbit IgG-HRP conjugate secondary antibody (1,1,000) for 2 h at room temperature. Protein bands were detected following incubation with a chemiluminescence substrate (PierceTM ECL Western Blotting Substrate; ThermoFisher Scientific, USA). The membranes were then scanned on the iBright imager, and band intensities were normalized to total protein levels using ImageJ software (NIH, Bethesda, ML, USA).

#### 2.3.6 Immunostaining and confocal microscopy

Brain tissues were extracted and immersed in a 4% paraformaldehyde (PFA) solution in phosphate-buffered saline (PBS), undergoing fixation at 4°C for 24 h. The tissues were then equilibrated with 10% sucrose/20% EDTA and 20% sucrose/20% EDTA for 48 h, and subsequently embedded in optimal cutting temperature compound (OCT, Fisher HealthCare, USA). The whole brain was cut into 10-micron sections on a cryotome (Leica, Germany) and mounted onto poly-L-lysine pre-coated glass slides (Superfrost Plus; Thermo Fisher). Slides were rehydrated in PBS and washed three times with PBTD (1X PBS with 0.1% Tween-20 and 1% DMSO). Subsequently, sections were blocked for 3 h in 5% normal goat serum (NGS) in PBTD (e.g., blocking buffer) followed by overnight incubation with rabbit anti-PSD-95 (1:300, Abcam, USA) or rabbit anti-gephyrin (1:800, Abcam, USA) antibody at 4°C in a humid chamber. On the next day, sections were washed three times with PBTD and incubated with Alexa Fluor® 488 secondary antibody (Thermo Fisher Scientific, USA) in PBTD for 2 h at room temperature. Optic tectum and telencephalon regions were imaged using a Zeiss Cell Observer Spinning disk inverted microscope equipped with a Yokogawa CSU-x1 confocal scanner and a Plan-Apochromat 63x/1.4 NA oil objective. PSD-95 and gephyrin puncta were quantified using the “Analyze Particles” function in ImageJ/Fiji, with puncta size ranging between 0.012 μm^2^ and 3.2 μm^2^. The density of PSD-95 and gephyrin signal in the optic tectum and telencephalon was calculated by dividing the total number of PSD-95 or gephyrin puncta by the surface area of the corresponding regions.

#### 2.3.7 Statistical analysis

Statistical analyses utilized SPSS software (version 23.0, IBM SPSS Inc., USA), and data were presented as mean ± S.E.M. unless stated otherwise. Normality and homogeneity of variances were assessed using the Kolmogorov–Smirnov one-sample test and Levene’s test, respectively. The potential impact of *kcc2a* KO on zebrafish behavior was assessed through an Independent Sample t-test, aiming to compare the responses between the control and mutant zebrafish groups. Variations in behavioral outcomes, mRNA abundance, and biochemical parameters among groups treated with pharmaceuticals were assessed using one-way ANOVA, followed by Tukey’s *post hoc* test for pairwise comparisons. Gene expression data underwent log transformation for variance stabilization. In cases of severe heteroscedasticity, Welch’s test with the Games–Howell *post hoc* test was employed. The arcsine square root transformation was performed on percentage data, where appropriate. The alpha level was set at 0.05.

## 3 Results

### 3.1 KCC2 dysregulation and socio-cognitive abnormalities in zebrafish larvae

Previous studies in rodents showed that homozygous KCC2^−/−^ mice and mice lacking *kcc2b* die after birth (Woo et al., 2002). To avoid this perinatal lethality, we decided to target *kcc2a* which is the dominant KCC2 isoform in the immature nervous system and possess similar levels of Cl^−^ transport function compared to KCC2b (Uvarov et al., 2007). To elucidate the molecular function of KCC2a in the zebrafish brain, we simultaneously targeted three exons of *kcc2a* using a novel CRISPR/Cas9-mediated genome editing strategy. The Sanger sequencing results revealed a disrupted nucleotide sequence in exon 2 of *kcc2a* in the F0 generation (Supplementary Figure S1), suggesting possible impairment of its gene function.

**Figure 1:**
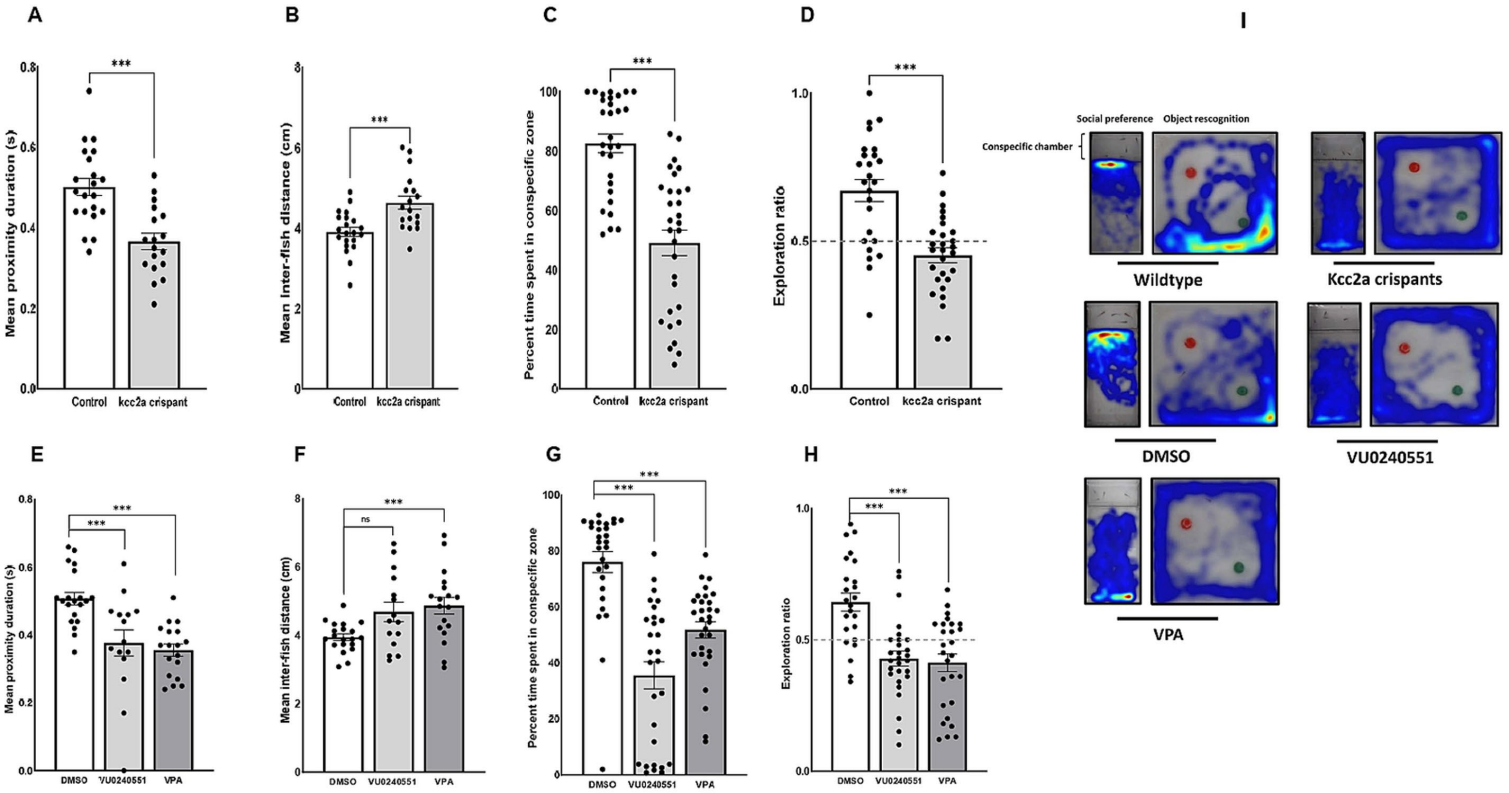
Alterations in social behaviors and cognitive performance in *kcc2a* crispants. Changes in shoaling behavior is shown as **(A)** mean proximity duration and **(B)** mean inter-fish distance (*n* = 4–7). Social preference is shown as **(C)** the percentage of time spent in the conspecific zone (*n* = 29–30), and **(D)** object recognition memory represented by exploration ratio (*n* = 25–28). The figure also depicts changes in (**E,F**) shoaling behavior (*n* = 15–20), **(G)** social preference (28–30), and **(H)** object recognition memory (*n* = 24–28) in fish exposed to VU 0240551 (a KCC2 blocker) and valproic acid (VPA). **(I)** Representative heat map of zebrafish larvae in social preference and object recognition tasks. The asterisks above data bars represent a significant difference vs. the control group at * *p* < 0.05, ** *p* < 0.01, and *** 0.001. “ns” indicates not significant (*p* > 0.05).

CRISPR-injected F0 embryos (so-called *kcc2a* crispants) and zebrafish larvae treated with the selective KCC2 inhibitor VU0240551 and VPA exhibited grossly a normal phenotype and locomotion, remained viable, and survived into adulthood. However, *Kcc2a* crispants displayed altered social behaviors. As shown in Figure 1A,B, time spent in proximity and the inter-individual distance were significantly reduced and increased, respectively (both *p* ≤ 0.001). Interestingly, the same scenario was observed in fish exposed to VPA (Figures 1E,F; *p* ≤ 0.001 and *p* ≤ 0.005). While there was a significant decrease in time spent in proximity in larvae exposed to KCC2 blocker (*p* ≤ 0.002), the change in inter-individual distance among subjects was statistically indistinguishable from the control group (*p* = 0.063). The results also revealed significant changes in social preference in *Kcc2a* crispants and treated fish (Figure 1C). F0 *kcc2a* KO fish exhibited a reduced social preference as shown by a decrease in the percentage of total time spent in the conspecific side (*p* ≤ 0.001). Likewise, a significant decrease in the total time spent in conspecific zone was observed in zebrafish larvae exposed to VPA and VU0240551 (Figure 1G; both p ≤ 0.001). In addition to social behaviors, cognitive performance in zebrafish larvae was assessed employing a widely used object recognition task. Our results revealed that F0 *kcc2a* KO zebrafish spent significantly less time exploring the novel object compared to the control group (i.e., decreases in exploration ratio) (Figure 1D; *p* ≤ 0.001) indicating memory impairment in *kcc2a* crispants. Likewise, embryonic exposure to VU0240551 and VPA recapitulated the memory deficits observed in F0 *kcc2a* knockouts (Figure 1H; both *p* ≤ 0.001).

### 3.2 Alterations in transcription of molecular markers of excitation/inhibition balance

**Figure 2:**
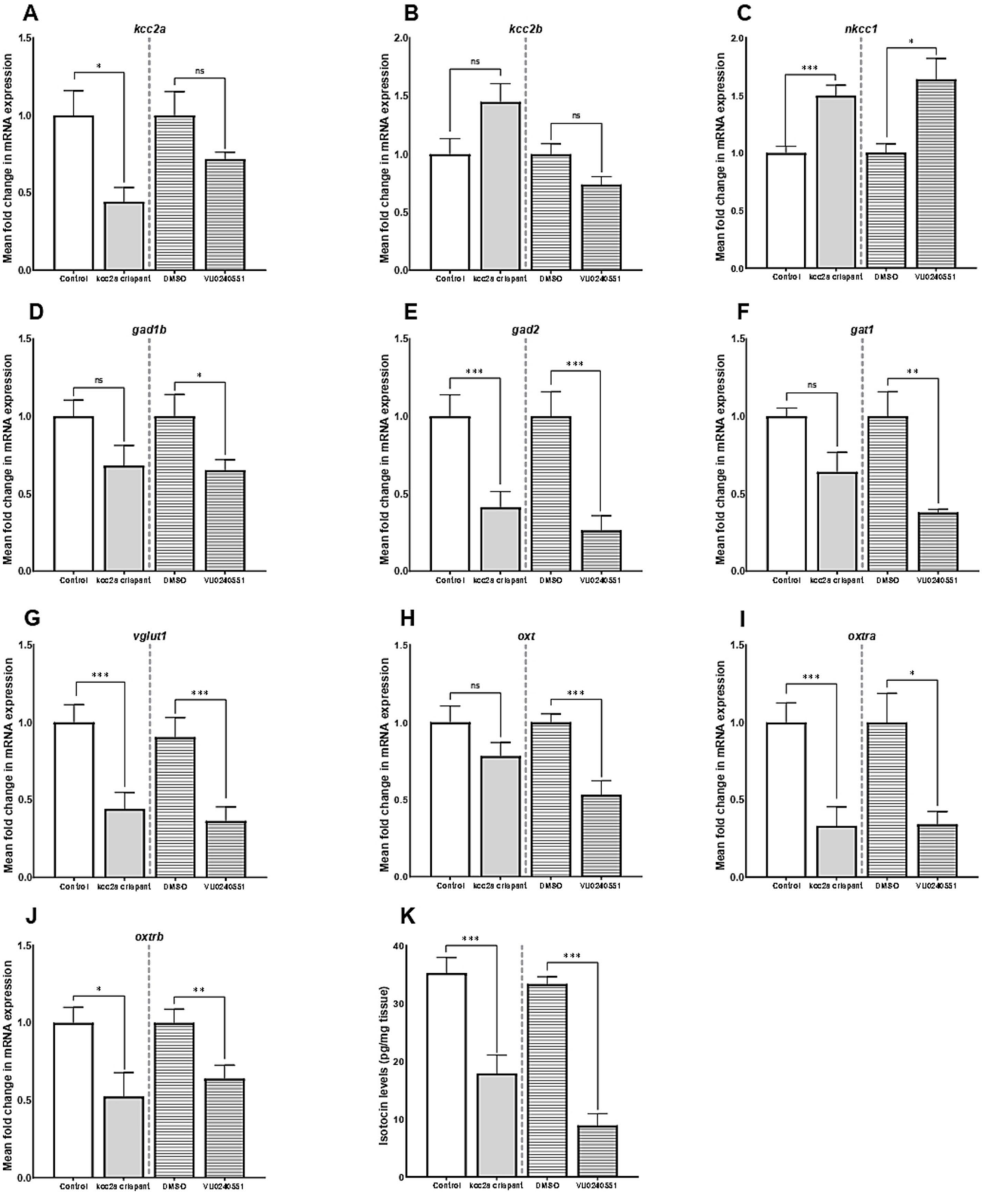
The mean fold change in the mRNA expression level of **(A)** *kcc2a* (*slc12a5a*), **(B)** *kcc2b* (*slc12a5b*), **(C)** *nkcc1* (*slc12a2*), **(D)** *gad1b* (glutamate decarboxylase 1b), **(E)** *gad2* (glutamate decarboxylase 2), **(F)** *gat1* (GABA transporter 1, *slc6a1a*), **(G)** *vglut1* (vesicular glutamate transporter 1, *slc17a7a*), **(H)** *oxt,* **(I)** *oxtra,* and **(J)** *oxtrb* genes (*n* = 5–7). **(K)** The whole-brain oxytocin (isotocin) levels between different experimental conditions (control vs. *kcc2a* crispants and DMSO-treated fish vs. KCC2 blocker VU 0240551, *n* = 4–6). The asterisks above data bars represent a significant difference vs. the control group at *p < 0.05, **p < 0.01, and ***p < 0.001. “ns” indicates not significant (*p* > 0.05).

Our results further revealed an alteration in expression of several genes crucial for establishment and maintenance of E/I balance in the CNS. As shown in Figures 2A,B, there was a significant decrease in the mRNA expression level of *kcc2a* (*slc12a5a*, *p* ≤ 0.014), while the levels of *kcc2b* (*slc12a5b*) showed a slight increase compared to the control fish, although the difference was not statistically significant (*p* = 0.052). There was also a significant increase in the mRNA expression levels of *nkcc1* (*slc12a2*) in *kcc2a* crispants compared to the control fish (Figure 2C; p ≤ 0.001). Although there was an apparent decline in the expression levels of glutamate decarboxylase 1b (*gad1b*), this difference was not statistically significant compared to the control (Figure 2D; *p* = 0.08). Furthermore, CRISPR-Cas9-mediated knockout of *kcc2a* gene led a significant decrease in the transcript levels of glutamate decarboxylase 2 (*gad2*, *p* ≤ 0.007), GABA transporter 1 (*gat1*, *slc6a1a*, *p* = 0.018), and vesicular glutamate transporter 1 (*vglut1*, *slc17a7a*, *p* = 0.004), known as molecular markers of GABAergic and glutamatergic systems (Figures 2E–G). A significant decrease in the transcript levels of both oxytocin receptor (*oxtra* and *oxtrb*, also known as isotocin receptor) was observed in *kcc2a* crispants (Figures 2I,J; *p* ≤ 0.003 and *p* = 0.022), while the mRNA levels of *oxt* gene remained unchanged compared to the control fish (Figure 2H; *p* = 0.13).

Embryonic exposure to the KCC2 blocker (VU0240551) also brought about changes in the transcript levels of several genes in the zebrafish brain. Our results depict a significant increase in the mRNA levels *nkcc1* (*slc12a2*, *p* = 0.024), while the transcript levels of both KCC2 isoforms (*slc12a5a* and *slc12a5b*) remained unchanged (Figures 2A–C; *p* = 0.53 and *p* = 0.12). Administration of the KCC2 blocker significantly decreased the mRNA levels of *gad1b* (Figure 2D; *p* = 0.04), *gad2* (Figure 2E; *p* = 0.007), *gat1* (Figure 2F; *p* = 0.003), and *vglut1* (Figure 2G; *p* = 0.004) in the brain of zebrafish larvae. In the same vein, we found a significant reduction in the transcript abundance of *oxt* (*p* ≤ 0.003) and its receptors *oxtra* (*p* = 0.022) and *oxtrb* (*p* ≤ 0.001) compared to the control group (Figures 2H–J).

As Figure 2K depicts, there was a significant reduction in whole-brain OXT protein levels (isotocin) in *kcc2a* crispants compared to the control fish (*p* ≤ 0.003). Likewise, a marked decrease in whole-brain OXT protein levels was observed in zebrafish larva embryonically exposed to the KCC2 blocker (VU0240551) compared to controls (DMSO, *p* ≤ 0.001).

### 3.3 Inhibitory/excitatory synaptic balance in KCC2a KO larvae

**Figure 3:**
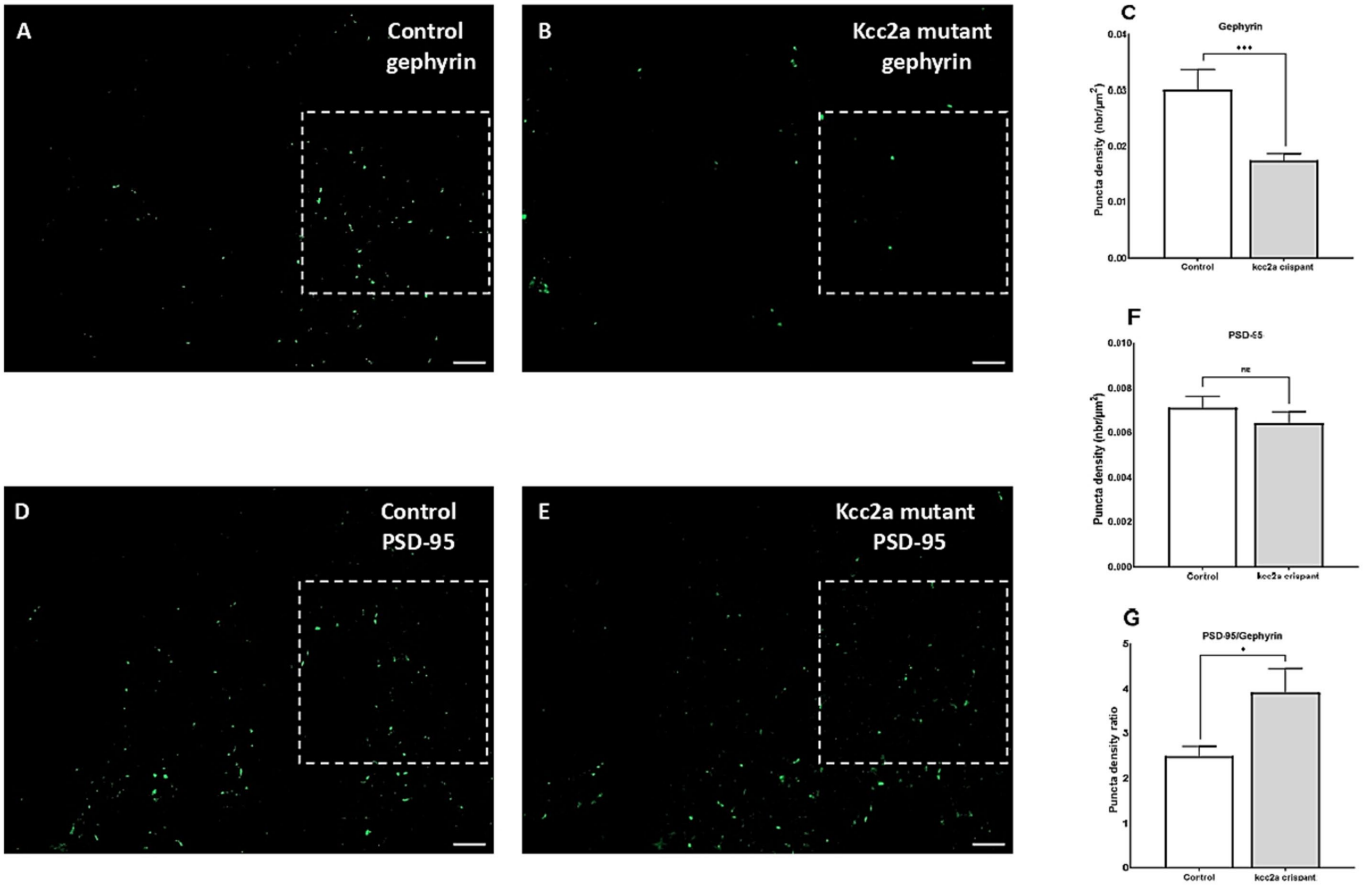
Defects of excitatory/inhibitory balance in the *kcc2a* KO larvae. 10 μm coronal sections of 21 dpf **(A)** control (*N* = 4, *n* = 12), **(B)** *kcc2a* crispants (*N* = 3, *n* = 9) stained with gephyrin, an inhibitory post-synaptic scaffolding protein and **(C)** quantification of gephyrin puncta density. **(D)** Coronal sections of 21 dpf control (*N* = 3, *n* = 9) larvae and **(E)** *kcc2a* crispants stained with PSD-95, an excitatory post-synaptic scaffolding protein and **(F)** quantification of PSD-95 puncta density. **(G)** PSD-95/gephyrin puncta density ratio. Scale bar 10 μm. N, number of larvae; n, number of sections. The asterisks above data bars represent a significant difference vs. the control group at *p < 0.05, **p < 0.01, and ***p < 0.001. “ns” indicates not significant (*p* > 0.05).

As shown in Figure 3, quantification of the two post-synaptic populations showed a marked decrease in gephyrin labeling (*p* ≤ 0.009, Figures 3A–C), while PSD-95 puncta density remained unchanged (*p* = 0.35, Figures 3D–F). This led to a significant increase in the ratio of excitatory to inhibitory neurons compared to the control (i.e., an increase in PSD-95/gephyrin puncta density ratio, Figure 3G; *p* ≤ 0.035).

### 3.4 BDNF expression in KCC2a KO larvae

**Figure 4:**
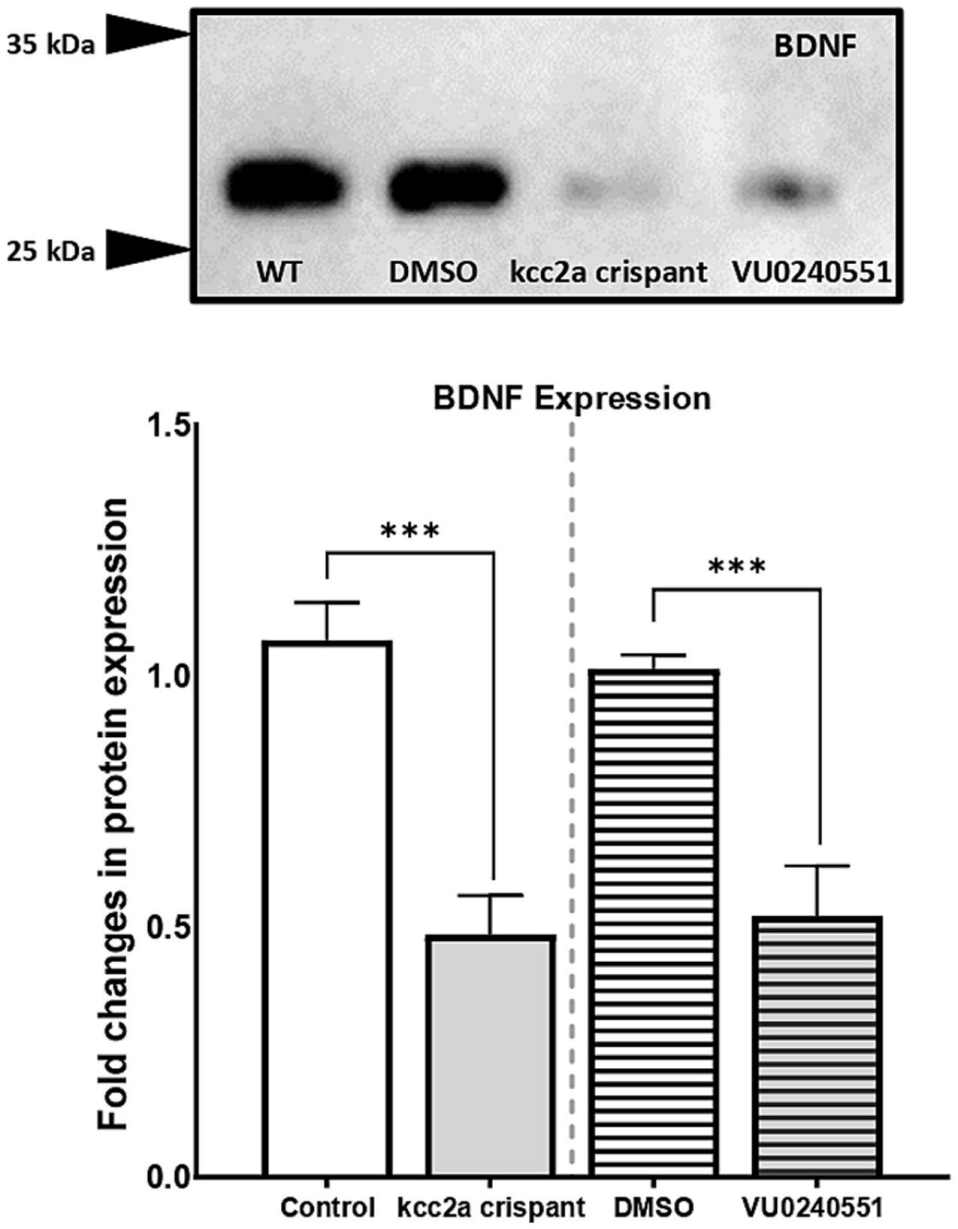
**(A)** Western blot analysis and **(B)** quantification of brain-derived neurotrophic factor (BDNF) protein levels in the zebrafish whole-brain (*n* = 3). Protein band intensities were normalized to total protein levels obtained via Ponceau staining represented as relative difference between experimental groups. The asterisks above data bars represent a significant difference vs. the control group at *p < 0.05, **p < 0.01, and ***p < 0.001. “ns” indicates not significant (*p* > 0.05).

The lack of a dependable antibody precluded the possibility of validating KCC2 protein levels in mutant zebrafish through western blot analysis. Therefore, we sought an alternative approach by utilizing a specific antibody targeting BDNF in our study. BDNF is the most well-studied modulator of KCC2 activation in immature neurons. In the developing brain, BDNF can increase KCC2 activation by regulating its localization at the membrane (Khirug et al., 2010; Puskarjov et al., 2014). Western blot analysis of brain samples from control and *Kcc2a* crispants revealed a significant change in BDNF expression (Figure 4). This change was attributed to a significant decrease (*p* = 0.005) in the expression of BDNF in *Kcc2a* crispants compared to the control. Likewise, there was a significant reduction (*p* = 0.009) in BDNF expression in the brain of zebrafish larvae embryonically exposed to the KCC2 blocker VU0240551.

### 3.5 Pharmacological rescue of behavioral abnormalities induced by KCC2 knockout

**Figure 5:**
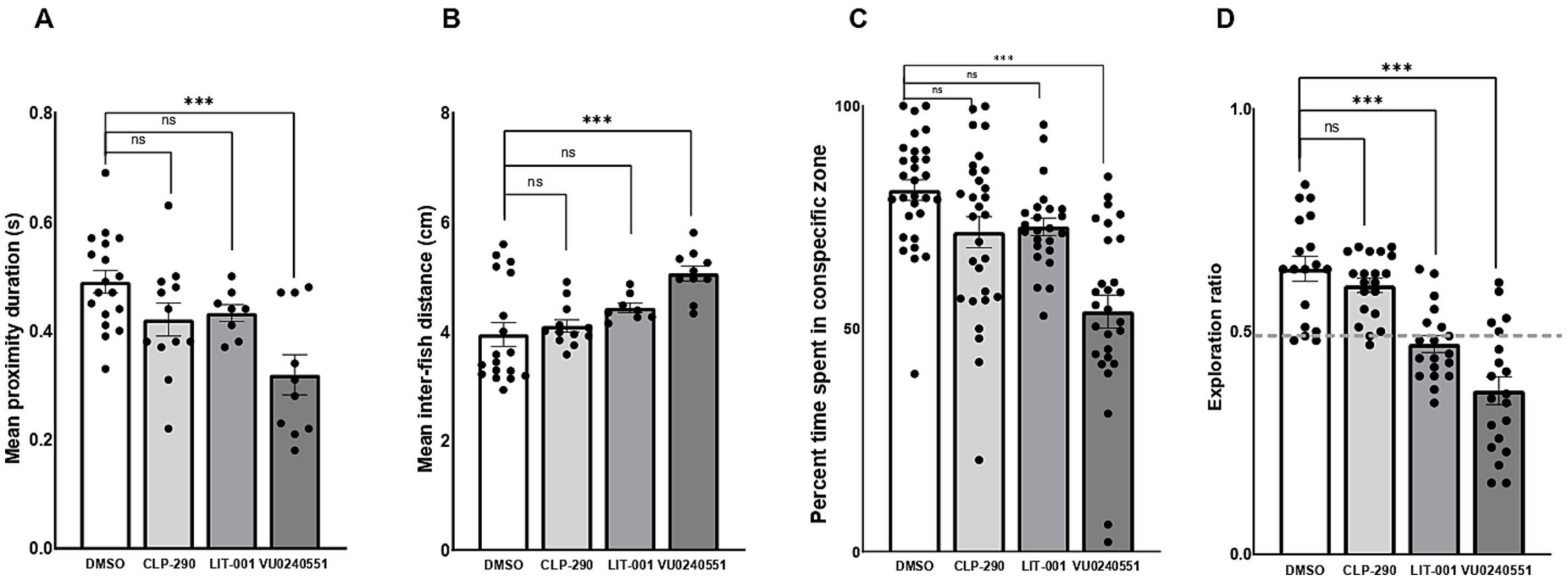
Effects of CLP-290, LIT-001, and VU 0240551 on social deficits, measured by time spent in proximity to shoal members **(A)**, inter-individual distance **(B)**, time spent in the conspecific zone **(C)**, and memory impairment **(D)** in kcc2a crispants. Asterisks above the data bars indicate significant differences compared to the control group: *p < 0.05, **p < 0.01, and ***p < 0.001. “ns” indicates not significant (*p* > 0.05).

We then explored the therapeutic potential of postnatal exposure to several novel compounds aimed at mitigating ASD-like behaviors in F0 *kcc2a* knockout zebrafish at 21 dpf. Our results demonstrated that a 48-h exposure to CLP-290 and LIT-001 successfully alleviated social abnormalities in F0 *kcc2a* KO zebrafish larvae (Figures 5A,B). This was evident in the comparison of time spent in proximity (*p* = 0.25 and *p* = 0.12) and inter-individual distance with DMSO-treated fish (*p* = 0.9 and p = 0.2). However, F0 *kcc2a* KO zebrafish larvae exposed to KCC2 blocker (VU0240551) showed decreased and increased time spent in proximity and inter-individual distance compared to the fish exposed to DMSO (Figures 5A,B; both *p* ≤ 0.001). Likewise, the postnatal administration of CLP-290 and LIT-001 restored diminished social preference in F0 *kcc2*a KO zebrafish larvae (all *p* ≤ 0.86, Figure 5C). Conversely, mutants exposed to the KCC2 antagonist (VU0240551) exhibited a significant decrease in the time spent with shoalmates compared to DMSO fish (*p* ≤ 0.003). Furthermore, postnatal administration of CLP-290 alleviated memory deficits in F0 *kcc2* KO zebrafish larvae when compared to DMSO-treated fish (Figure 5D; both *p* ≤ 0.84). However, postnatal exposure to either oxytocin receptor agonist (LIT-001) or KCC2 blocker (VU0240551) failed to rescue cognitive performance in *Kcc2a* crispants in the object recognition task compared to DMSO control treatment (both *p* ≤ 0.001).

## 4 Discussion

KCC2 is a vital neuron-specific K^+^/Cl^−^ cotransporter that sets the strength and polarity of GABAergic currents during neuronal maturation. Dysregulation in its expression and function has been linked to the pathogenesis of various neurological disorders, including epilepsy, Rett Syndrome, schizophrenia, and ASD (Hyde et al., 2011; Merner et al., 2015; Tomita et al., 2023). In this study, we employed a novel synthetic CRISPR-Cas9-based mutagenesis approach for generating biallelic F0 zebrafish knockouts to study the functional importance of the *kcc2a* gene in the development of socio-cognitive functions in the zebrafish larvae. The *kcc2a* crispants displayed normal morphology, body length, and motor function and survived into adulthood as reported previously (Stödberg et al., 2015). However, the results of behavioral assessment revealed marked socio-cognitive deficits in F0 mutants. The F0 *kcc2a* KO zebrafish larvae displayed abnormal social behaviors which was evidenced by a reduction in shoaling behavior and social preference. Moreover, *kcc2a* KO fish exhibited impaired object recognition memory, as documented by a decrease in novel object exploration ratio. Embryonic administration of the KCC2 blocker VU0240551 also reduced shoaling behavior, social preference, and compromised recognition memory in 21 dpf zebrafish larvae. This suggests a shared mechanistic pathway affected by both genetic and pharmacological perturbations, reinforcing the critical involvement of KCC2 in shaping fundamental aspects of socio-cognitive functions in zebrafish larvae. Our findings align with prior studies indicating that mutations or premature onset of KCC2 function during early developmental stages lead to enduring abnormalities in social behavior and memory. For example, disrupting the phosphorylation process impeded the postnatal establishment of KCC2 function, consequently yielding enduring abnormalities in social behavior and memory retention in mice (Moore et al., 2019). Tamoxifen-induced conditional deletion of KCC2 in the glutamatergic neurons resulted in spatial and nonspatial learning impairments in 3 months old mice (Kreis et al., 2023). In another study, however, KCC2b heterozygous knockout mice (KCC2^+/−^) demonstrated enhanced social dominance behaviors and elevated amplitude of spontaneous postsynaptic currents within the medial prefrontal cortex (PFC), a crucial region implicated in governing social hierarchy and dominance behaviors (Anacker et al., 2019). These suggest that any departure from a precise balance between excitation and inhibition may contribute to aberrant behavioral manifestations. Intriguingly, these behavioral abnormalities mirrored those observed in zebrafish larvae embryonically exposed to VPA, a well-established inducer of ASD-like traits, suggesting a parallel between KCC2 dysfunction and established models of ASD. Prenatal exposure to VPA also decreased protein levels of KCC2 and resulted in impaired spatial memory, limited exploration, increased anxiety, and reduced sociability in the model group (Li et al., 2017). In a more recent study, Haratizadeh et al. (2023) have shown that prenatal VPA exposure increased repetitive/stereotyped movements and impaired object recognition memory and social behaviors in adult rats. These deficits were linked to the reduction of KCC2, causing subsequent disruption in E/I balance. Taken together, these findings underscore the pivotal role of KCC2 in socio-cognitive functions, where genetic or pharmacological disruptions induce lasting abnormalities resembling ASD models, emphasizing the significance of excitation-inhibition balance.

Transcriptomic analyses using ddPCR unveiled marked changes in the expression levels of key genes involved in maintaining E/I balance. CRISPR-Cas9-mediated knockout of *kcc2a* led to a reduction in the transcript abundance of the *kcc2a* gene, with a concurrent elevation in *kcc2b* mRNA levels. Given the abundant expression of both *kcc2a* and *kcc2b* isoforms with their shared ability for chloride extrusion in neonatal animals, the absence of one isoform seems to be partially compensated for by the other as suggested previously (Markkanen et al., 2014). Moreover, the downregulation of *kcc2a* was accompanied by an upregulation in the expression of *nkcc1*, indicating a possible disruption in chloride homeostasis. This *nkcc1* upregulation might be attributed to the imperative need for maintaining a low intracellular chloride concentration ([Cl^−^]i) within neurons, coupled with extracellular potassium ([K^+^]o) accumulation, likely stemming from prolonged downregulation of *kcc2a*. The *Kcc2a* KO also caused a marked reduction in the mRNA expression of key molecular markers associated with the GABAergic (*gad2* and *gat1*) and glutamatergic (VGLUT1) systems. The attenuated *gad2* expression, responsible for GABA synthesis, suggests a potential decline in inhibitory signaling, while the reduced VGLUT1 levels indicate compromised excitatory neurotransmission due to decreased glutamate release. Additionally, the decreased expression of *gat1* implies a potential prolongation of inhibitory signals. Collectively, these molecular alterations may contribute to an aberrant E/I balance and perturbed neural circuitry. Consistent observations in zebrafish larvae treated with the KCC2 blocker VU0240551 during embryonic development further substantiate the association between KCC2 dysfunction and E/I imbalance. Our findings are in agreement with previous studies in rodents reporting that KCC2 mutation and/or inhibition contributes to E/I imbalance in mice and rats (Gauvain et al., 2011; Engberink et al., 2018; Raol et al., 2020). Moreover, KCC2 deficits in mouse models of Rett syndrome and ASD have been linked to the altered the polarity of GABAergic inhibition in cortical neurons (Banerjee et al., 2016). Using KCC2-mutant mice, Pisella et al. (2019) have also reported an altered GABAergic inhibition and increased glutamate/GABA synaptic ratio in cortical and hippocampal pyramidal neurons, reinforcing the role of KCC2 in regulating E/I balance across different neurological contexts.

The E/I imbalance in ASD is associated with increased excitation or reduced inhibition, leading to a higher excitatory-to-inhibitory ratio. Individuals with ASD often show decreased GABAergic signaling, which is theorized to contribute to cognitive deficits, repetitive behaviors, and abnormal social behaviors (Rubenstein and Merzenich, 2003). The measurement of PSD-95 and gephyrin serves as crucial indicators of synaptic function, particularly at excitatory and inhibitory synapses, respectively. PSD-95, located at post-synaptic density of excitatory synapses, plays a vital role in stabilizing synaptic contacts and in synaptic maturation for glutamate receptors. On the other hand, gephyrin, a critical component at inhibitory synapses, interacts with GABA_A_ and glycine receptors, facilitating inhibitory neurotransmission (Chen et al., 2014). In this study, we illustrated that the knockout or inhibition of *kcc2a* resulted in a reduction of inhibitory post-synaptic terminals, consequently leading to an increased ratio of excitatory to inhibitory neurons. Gephyrin directly interacts with KCC2 to regulate its surface expression and function in cortical neurons. KCC2 knockdown has been shown to reduce gephyrin protein in primary cultures of spinal cord neurons (Schwale et al., 2016). Moreover, the reduction in gephyrin may be a consequence of disrupted inhibitory neurotransmission due to the absence or reduction of functional *kcc2a* transporter. On the other hand, the unchanged levels of PSD-95 could be a result of homeostatic mechanisms attempting to stabilize excitatory synapses in response to the disruption of inhibitory synapses. Overall, these findings reveal that early life disruption of *kcc2a* may reduce the efficacy of inhibitory neurotransmission and increase neuronal excitability in the brain of zebrafish larvae.

There is conclusive evidence that E/I imbalance affects the function of neural circuits involved in social cognition and emotional regulation leading to altered information processing and transmission in the brain (Yizhar et al., 2011). Disruptions in inhibitory signaling, particularly in the GABAergic system, may also contribute to aberrant neural responses to social stimuli, leading to difficulties in interpreting social cues and engaging in appropriate social behaviors. On the other hand, excitatory dysfunction involves irregularities in the functioning of excitatory neurotransmitters such as glutamate leading to abnormal neuronal activation. This disruption can result in hyperactivity or hypoactivity within neural networks associated with social cognition and memory, ultimately affecting the encoding, consolidation, and retrieval of social information. Deleterious effects of E/I imbalance on neural processes extends to the modulation of key neuromodulators, notably OXT. Oxytocin, a hypothalamic neuropeptide, stands as a pivotal regulator of diverse social behaviors across various species. During early brain development, oxytocin and its receptor play fundamental roles in orchestrating the postnatal shift of neuronal GABA neurotransmission from an excitatory to an inhibitory state. Leonzino et al. (2016) has illustrated that the oxytocinergic signaling actively influences the functional dynamics of KCC2 by facilitating its phosphorylation and subsequent insertion/stabilization in the plasma membrane. Gigliucci et al. (2022) also reported a concomitant reduction in the KCC2 and OXTR expression in a mouse model of Rett syndrome. Additionally, it has been demonstrated that the down-regulation of KCC2 induced by lipopolysaccharide (LPS) led to a reduction in OXTR mRNA levels, highlighting a mutual relationship between oxytocin and chloride homeostasis (Tomita et al., 2022). The results of the present study also demonstrated a decrease in the mRNA expression of OXTR genes and whole-brain OXT levels, a finding that was further corroborated through the administration of the KCC2 blocker VU0240551. Together, these findings suggest that the absence of physiological upregulation of the chloride transporter KCC2 during early brain development can result in aberrant E/I balance, which in turn, compromises the firing rates of OXT-producing neurons, the dynamics of OXT release, and the responsiveness of OXTRs, collectively contributing to a decrease in oxytocin expression levels.

Signaling via BDNF and its receptor, tropomycin receptor kinase B (TrkB) is one of the most critical regulators of glutamatergic and GABAergic synapse development (Cohen-Cory et al., 2010). On the other hand, KCC2 regulates dendritic spine formation in hippocampal and cortical neurons in a BDNF-dependent manner (Awad et al., 2018). KCC2 has also been shown to play a key role in the regulation of BDNF–TrkB in rats (Zhang et al., 2023). The deficiency of MECP2 in Rett syndrome has also been associated with the downregulation of BDNF, while the overexpression of KCC2 ameliorated the phenotype (Abuhatzira et al., 2007; Tang et al., 2016). In this study, we found that either genetic or pharmacological disruption of *kcc2a* resulted in a marked decrease in BDNF levels in the zebrafish brain, reinforcing the compelling connection between KCC2 and BDNF. It is plausible that the loss of KCC2 function disrupts the balance between excitatory and inhibitory signaling, leading to depolarizing GABAergic responses and heightened neuronal excitability, which impairs activity-dependent BDNF transcription (Porcher et al., 2018). KCC2 dysfunction may also alter intracellular calcium homeostasis, which is critical for the regulation of BDNF expression via calcium-dependent transcription factors such as cAMP-response element binding protein CREB (Shieh and Ghosh, 1999). Disruption of calcium signaling can, therefore, reduce BDNF expression and impair synaptic plasticity. Furthermore, the loss of KCC2 function may compromise the integrity of BDNF/TrkB signaling pathways, thereby diminishing BDNF-mediated neurotrophic support and synaptic efficacy (Rivera et al., 2002). While these mechanisms offer plausible explanations, the precise molecular pathways linking KCC2 disruption to decreased BDNF expression remain unclear and warrant further investigation. BDNF plays a critical role in synaptic plasticity and neurotransmitter release (Lu et al., 2014). Decreased BDNF expression has been associated with a range of neurological disorders, including schizophrenia (Nieto et al., 2013), Alzheimer’s disease (Lee et al., 2005), and ASD (Ricci et al., 2013). In zebrafish, CRISPR/Cas9-mediated knockout of the BDNF gene resulted in learning deficits and abnormal social behaviors (Lucon-Xiccato et al., 2022; Lucon-Xiccato et al., 2023). Therefore, the observed reduction in BDNF levels following *kcc2a* knockout is likely to contribute to synaptic dysfunction, thereby affecting signal transmission in brain regions essential for memory and social behavior.

While the behavioral and molecular consequences of KCC2a disruption were profound, our study went a step further to explore the therapeutic potential of pharmaceuticals that affect the expression and function of KCC2. In recent years, enhancing KCC2 function has emerged as a promising therapeutic target for the treatment of a wide range of neurological disorders (Lam et al., 2023; Tomita et al., 2023). For example, restoring KCC2 using short-term CLP-290 treatment, successfully alleviated spatial memory deficits and improved social function in a mouse model of Alzheimer’s disease (Keramidis et al., 2023). Likewise, we found that 2 days of exposure to CLP-290 rescued both recognition memory and social abnormalities in *kcc2a* KO zebrafish. Intranasal OXT has long been known as a potential treatment for addressing socio-cognitive dysfunctions in various brain disorders, such as ASD (Keech et al., 2018). The potential therapeutic benefits of oxytocinergic drugs have recently been correlated with their capacity to boost the expression and activity of KCC2. For instance, OXT treatment has been shown to restore KCC2 expression and E/I balance in a mouse model of Rett syndrome (Gigliucci et al., 2022). Neonatal subcutaneous OXT administration improved social memory deficits and reversed KCC2 dysfunction in hippocampal GABAergic activity in a mouse model of ASD (Bertoni et al., 2021). In another study, 3 days continuous intrathecal infusion of OXT restored the expression levels of KCC2 in the spinal dorsal horn caused by nerve injury (Ba et al., 2022). Furthermore, Rahmati-Holasoo et al. (2023) have recently shown that 48 h OXT treatment during the larval stage improved ASD-like behaviors in a VPA zebrafish model. The findings of this study align with previous research, affirming that the administration of LIT-001 rescued social behaviors in *kcc2a* crispants. However, the lack of LIT-001’s effect on object recognition memory indicates that LIT-001 may modulate neural circuits or pathways associated with social behaviors, while its influence on cognitive functions, particularly memory, may be limited or absent. These observations underscore the intricate nature of neurobehavioral modulation and highlight the importance of delineating specific cognitive domains when evaluating the effects of pharmacological interventions. Further investigations into the molecular and neural mechanisms targeted by LIT-001 can provide deeper insights into its functional profile and potential therapeutic applications.

In conclusion, our study, for the first time, shed light on the critical role of KCC2 in shaping socio-cognitive functions in zebrafish larvae, with disruptions leading to long-lasting behavioral abnormalities. The observed abnormalities in behavioral outcomes, shared between genetic knockout and pharmacological perturbations, underscore the pivotal involvement of KCC2 in the developmental regulation of socio-cognitive functions. Molecular analyses demonstrated altered expression of key genes associated with the GABAergic and glutamatergic systems, contributing to an aberrant E/I balance and perturbed neural circuitry. Notably, these disruptions mirrored the behavioral abnormalities observed in established models of ASD (VPA-induced ASD), emphasizing the significance of excitation-inhibition balance in socio-cognitive functions. Furthermore, our exploration of therapeutic interventions targeting KCC2, such as CLP290 and LIT-001, demonstrated promising outcomes in rescuing memory and social abnormalities. However, the selective effects of LIT-001 on social behaviors, not memory, underscore the complexity of neurobehavioral modulation. These findings underscore the potential therapeutic avenues for KCC2-related neurological disorders, emphasizing the need for further investigations into the molecular and neural mechanisms underlying these effects.

## Data Availability

The datasets presented in this study can be found in online repositories. The names of the repository/repositories and accession number(s) can be found in the article/Supplementary material.
