# Supplementary material for: Unraveling the socio-cognitive consequences of KCC2 disruption in zebrafish: implications for neurodevelopmental disorders and therapeutic interventions
Source: Front Mol Neurosci. 2024 Oct 14;17:1483238. doi: 10.3389/fnmol.2024.1483238 (PMC11513385; doi:10.3389/fnmol.2024.1483238)
Supplement: Supplementary file 1 [file Data_Sheet_1.docx]

**Supporting Information**

**Unraveling the Socio-Cognitive Consequences of KCC2 Disruption in Zebrafish: Implications for Neurodevelopmental Disorders and Therapeutic Interventions**

Mohammad Naderi*, Thi My Nhi Nguyen, Christopher Pompili, Raymond WM Kwong

Department of Biology, York University, Toronto, ON, Canada, M3J 1P3

**Table of Contents:**

**Number of pages: 4**

**Number of figures: 1**

**Number of tables: 3**

*** Corresponding Author: Mohammad Naderi (naderimohd@gmail.com/mon86@yorku.ca)**

**
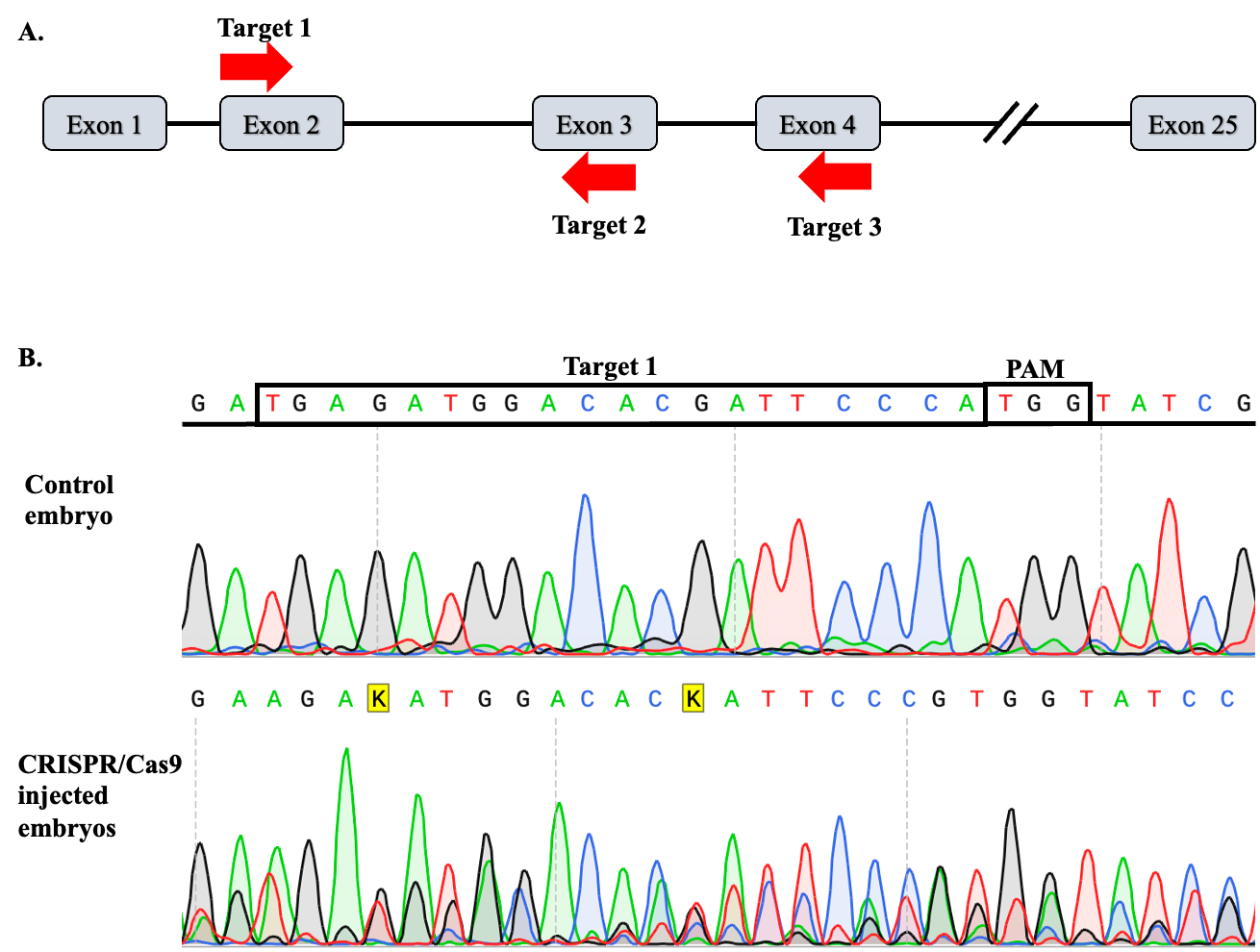
**

**Figure S1.** Generation of kcc2a knockout zebrafish mutants using Alt-R^TM^ CRISPR/Cas9 genome editing. **(A)** Schematic illustration of the application of Alt-R^TM^ CRISPR/Cas9 technology for rapid generation of F0 mutants to screen *kcc2a* function. Three loci were targeted simultaneously at different exons of *kcc2a* gene. **(B)** Representative Sanger sequencing chromatograms from DNA of control and CRISPR/Cas9-injected embryos.

**Table S1.** crRNAs and sgRNA target sites, genomic locations, the protospacer adjacency motif (PAM) sequences, on-target scores, and off-target scores

| **Gene** | **Exon target** | **Target sequence** | **PAM** | **On-target score** | **Off-target score** | **sgRNA** |
| --- | --- | --- | --- | --- | --- | --- |
| Control 1 | N/A | TGAGATGGACACGATTCCCA | TGG | 83 | 91 | TGAGATGGACACGATTCCCAGUUUUAGAGCUAUGCU |
| Control 2 | N/A | TGAGGAATAGAATCACACCC | AGG | 72 | 89 | TGAGGAATAGAATCACACCCGUUUUAGAGCUAUGCU |
| Control 3 | N/A | CACGACGCCGTTCGTGGCAA | TGG | 51 | 98 | CACGACGCCGTTCGTGGCAAGUUUUAGAGCUAUGCU |
| slc12a5a | 2 | TGAGATGGACACGATTCCCA | TGG | 55 | 87 | UGAGAUGGACACGAUUCCCAGUUUUAGAGCUAUGCU |
| slc12a5a | 3 | TGAGGAATAGAATCACACCC | AGG | 81 | 92 | UGAGGAAUAGAAUCACACCCGUUUUAGAGCUAUGCU |
| slc12a5a | 4 | CACGACGCCGTTCGTGGCAA | TGG | 56 | 96 | CACGACGCCGUUCGUGGCAAGUUUUAGAGCUAUGCU |

The scores are obtained from the CRISPR-Cas9 gRNA design checker developed by IDT.

**Table S2.** Primer sets used for genotyping

| Target region | Forward primer | Reverse primer |
| --- | --- | --- |
| slc12a5a exon2 | AATAACTCGCCTTCTGGGCTT | AACCCTCATTGTTCTCCGCC |
| slc12a5a exon3 | CTCTCTGTGTGTTTCTAGGCTCC | AAAGTAGTGAGACGCAGCCTTTT |
| slc12a5a exon4 | TGGAAACCACGATGCCTTGTA | CTGTCTGGTCATTGCTCGGA |

| **Target Gene** | **Forward primer** | **Reverse primer** | **NCBI accession number** |
| --- | --- | --- | --- |
| *Rps18* | CCCTCGTCATCCCAGAGAAGT | CGCCTTCCAACACCCTTAATAG | NM_173234.1 |
| *Rpl13a* | GTATTTGGCTTTCCTCCGCA | ACCATGCGCTTTCTCTTGTC | NM_212784.1 |
| *oxt* | TTGGTCGGCTCTCCAGAAAC | CTGTTGGCCGGTTGATTGAC | NM_178291.2 |
| *oxtra* | TTCTCCGTGCAGATGTGGTC | TAGAGCGTGGAGCAGCAAAA | NM_001199370.1 |
| *oxtrb* | TCAGATGTGGTCCGCTTGGG | CATGGCACCCGTCCCTGAAT | NM_001199369.1 |
| *kcc2a* | ACCTGACCGAATGCGAGGAG | TTTTTGGGCGGACAGGTGGT | XP_021332993.1 |
| *kcc2b* | ACCAAGAATGAGCGGGAGAG | AATGAGCTGAACCTCCTCTTCA | NM_001302243.1 |
| *nkcc1* | TAACGGCGAGGAGTTGACCC | TTGGCCCACGATCCATGTCA | NM_001002080.1 |
| *gad1b* | CACCTGTGACTCCGTAAGTGT | TGCCCAGTTTCCTCGTGCAT | NM_194419.1 |
| *gad2* | CAAGAAGCATGACGTCTGGA | CTGCATCAGTCCCTCCTCTC | NM_001017708.2 |
| *gat1* | AAGGAGTGGGCTGGCTTATG | ACTGCTCTTGTATCGGTGGC | NM_001045287.2 |
| *vglut1* | TATGGGCTCTTCGCGTCAGG | CCCATTAGGACCGCCGACAT | NM_001098755.1 |

**Table S3.** Primer sets used for ddPCR validation of differentially expressed genes
